# Toward the Optimal Choice of Gelled Vehicles for Oral Drug Administration in Dysphagic Patients

**DOI:** 10.3390/pharmaceutics17020251

**Published:** 2025-02-14

**Authors:** Serena Logrippo, Roberta Ganzetti, Matteo Sestili, Diego Romano Perinelli, Marco Cespi, Giulia Bonacucina

**Affiliations:** 1 Hospital Pharmacy, Santa Maria della Stella Hospital, USL Umbria 2, 05018 Orvieto, Italy; 2 Hospital Pharmacy, Engles Profili Hospital, AST Ancona, 60044 Fabriano, Italy; 3 Hospital Pharmacy, Carlo Urbani Hospital, AST Ancona, 60035 Jesi, Italy; 4 Territorial Pharmaceutical Service, AST Ancona, 60035 Jesi, Italy; 5 School of Pharmacy, University of Camerino, CHIP Building via Madonna delle Carceri, 62032 Camerino, Italy; diego.perinelli@unicam.it (D.R.P.); giulia.bonacucina@unicam.it (G.B.)

**Keywords:** thickened beverages, pravastatin sodium, swallowing difficulties, complex viscosity, rheological moduli, drug dissolution

## Abstract

**Background/Objectives**: Thickened waters are commonly used for dysphagic patients to ensure hydration, facilitate safer swallowing, and administer oral therapies, yet their impact on drug dissolution remains unclear. This study aims to investigate how thickening agents, viscosity, and solid oral dosage form (SODF) formulations influence drug release in gelled vehicles. **Methods**: Twelve commercially available thickened waters, including both ready-to-use products and powders for extemporaneous preparation, were used to disperse crushed sodium pravastatin tablets. The resulting preparations were evaluated for their rheological properties and dissolution performance. **Results**: Thickened water products vary in consistency, with starch-based thickeners providing more consistent results than gum-based ones. Pravastatin release profiles closely matched the original tablets with starch thickeners, while gum-based thickeners showed greater variability, primarily influenced by viscosity. **Conclusions**: These findings emphasize the importance of selecting the appropriate thickening agent for controlling drug release in thickened water products, highlighting the need to balance patient compliance with the potential impact on drug release during product development.

## 1. Introduction

Thickened waters (also referred to as gelled waters or, more generally, thickened beverages) are water or other liquids (sometimes juices) that were modified by the addition of thickening agents to achieve a specific viscosity. From a regulatory perspective, thickened waters may be classified as food for special medical purposes (FSMPs) under EU Regulation No. 609/2013 [1], provided they are intended to meet the specific nutritional requirements of patients with impaired capacity to consume ordinary foodstuffs.

The modification of beverage consistency makes liquids easier and safer to swallow for individuals with swallowing difficulties, such as those suffering from dysphagia, who often struggle with certain consistencies, particularly solid foods and thin liquids [2,3]. For these individuals, thickened waters help ensure proper hydration while reducing the risk of aspiration and choking by providing a controlled consistency [4,5]. Several associations involved in the management, therapy, and support of dysphagic patients provided recommendations on using liquids with appropriate viscosity or consistency to reduce the risk of aspiration pneumonia [6]. Currently, the proper consistency of thickened waters is ensured by adherence to the NDD (National Dysphagia Diet) guidelines established by the American Dietetic Association and the National Dysphagia Diet Task Force [7], or to the IDDSI (International Dysphagia Diet Standardisation Initiative) recommendations, developed by an international multidisciplinary group (IDDSI Committee) supported by numerous International Reference Groups [8]. The market currently offers various thickened waters available in two forms: ready-to-use thickened liquids (RU_TL) and formulated powders designed for the extemporaneous preparation of thickened liquids (EP_TL). In both cases, the desired consistency is achieved through the use of thickening agents, which are polysaccharides belonging to the group of modified starches or gums. Among the starches, pregelatinized starches are commonly used for preparing thickened beverages, typically derived from corn starch, although alternatives, such as those sourced from potatoes, are also commercially available. On the other hand, the most commonly used gums include xanthan gum, guar gum, and other gum-like polysaccharides, such as carrageenan and pectin. In gum-based products, these agents are typically used in combination to achieve optimal consistency and functionality [9,10]. The use of liquids with appropriate consistency is the most suitable approach for dysphagic patients, except for those unable to swallow or in cases where oral nutrition does not ensure proper hydration. In such situations, an enteral nutrition strategy, such as administration via a nasogastric tube or percutaneous endoscopic gastrostomy, is required [11].

Thickened waters are used not only for hydration but also for administering the oral therapies when alternative dosage forms suitable for dysphagic, such as suppositories or transdermal patches, are not commercially available. A common practice in dysphagic patients is to crush tablets or open capsules, then disperse or dissolve their contents in modified-consistency beverages [10,12]. This practice can modify drug bioavailability by influencing the drug release process, due both to the manipulation of the solid oral dosage form (SODF) and the interaction of the dispersed powder with the thickened vehicle. While the effects of manipulating the SODF were extensively studied in the literature [12,13,14,15], the latter aspect requires further investigation and clarification. Currently, there are few studies focusing on this aspect. Manrique et al. reported a reduction in the release rate of several drugs when crushed tablets were dispersed in gum-based and starch-gum-based vehicles, hypothesizing that the effect was due to the consistency and/or structure of the carrier vehicles [16,17]. Similar findings was reported by Ilgaz et al., which demonstrated that the gelling vehicles significantly influences the drug’s dissolution rate, especially in thickened samples prepared with xanthan gum [18]. These studies highlight that the gelled vehicles affect the dissolution rate of the drugs. However, several aspects remain to be clarified. First, although all the studies suggest a relationship between the reduction in dissolution rate and viscosity, this correlation has never been conclusively proven. Additionally, the number of commercial thickened waters analyzed was limited, despite the wide variety of products available on the market. These include both ready-to-use thickened liquids (RU_TL), which may come in a single consistency or in multiple consistency grades, and formulated powders designed for the extemporaneous preparation of thickened liquids (EP_TL). Moreover, most commercial products are gum-based, containing thickening agents such as guar gum, xanthan gum, carrageenan, and pectin, typically used in blends. However, starch-based products are also available. The effect of the type of thickening agent (gum-based or starch-based) remains unclear, although some authors suggest that starch-based products have a lesser impact on the drug release rate. Finally, it remains to be clarified whether the reduction in the drug release rate is exclusively due to the gelled vehicles or if it can also be partially attributed to the amount of SODF added or their formulation (the presence of specific excipients).

This study aims to address existing knowledge gaps by clarifying how thickening agents (gum-based vs. starch-based), viscosity, and SODF formulation (including excipients) influence drug dissolution in gelled vehicles. To achieve this aim, four ready-to-use thickened liquids (one available in three different consistencies) and two formulated powders for extemporaneous preparation of thickened liquids (gum- and starch-based) were selected, for a total of twelve different gelled vehicles. Tablets of sodium pravastatin (PraNa) were chosen as the model drug, given their frequent use among the elderly population, who are most likely to experience swallowing difficulties or disorders. The selection was also motivated by the drug’s high water solubility and its availability as immediate-release tablets. Additionally, the availability of several equivalent medicinal products allows testing across different formulations, including both coated and uncoated tablets.

## 2. Materials and Methods

### 2.1. Materials

Uncoated 20 mg pravastatin sodium tablets from two different manufacturers (Pravastatina Pensa, Pensa Pharma S.p.A., Milano, Italy and Pravastatina ratiopharm, Teva Italia s.r.l., Milano, Italy) and coated 20 mg pravastatin sodium tablets (Pravastatina EG, EG S.p.A., Milano, Italy) were purchased from a local pharmacy. Pravastatin sodium powder was kindly provided by Teva Pharmaceutical Industries Ltd. (Debrecen, Hungary) through Angelini Pharma s.p.a. (Rome, Italy).

Thickened water sweet fruit taste, Gelly’Gel sweetened, and Hydra’Fruit grade 1, 2 and 3 (Nutrisens Medical, Chaponost, France), as well as Resource bevanda gelificata (Nestlé Italiana S.p.A., Assago, Italy), were all ready-to-use thickened liquids (RU_TL). Resource ThickenUp Clear and Resource ThickenUp (Nestlé Italiana S.p.A., Italy) were formulated powders for preparing thickened liquids (extemporaneous preparation of thickened liquids EP_TL). Both the RU_TL and EP_TL were purchased from a local pharmacy.

Table 1 provides a schematic summary of all the ready-to-use gelled waters and formulated powders used in this study, including their thickening agents, available grades, and abbreviations used in the manuscript.

Through the manuscript, pravastatin sodium is abbreviated as PraNa.

### 2.2. Preparation of Thickened Water with Formulated Powders

The extemporaneous preparation of thickened liquids was carried out according to the manufacturers’ instructions. A specified amount of powder (varying by product and the desired consistency grade) was dispersed in a defined volume of water to achieve three different consistencies for each product, nectar (Ne), honey (Ho), and pudding (Pu), as classified by the NDD system [7]. The exact quantities of powder and water used in the preparations are detailed in Table 2.

### 2.3. Weight and Content Uniformity for PraNa Tablets

All the PraNa tablets were characterized in terms of weight and PraNa content. The weight of the tablets was determined using a three-decimal digit balance on a sample of 10 tablets.

The content uniformity was evaluated using a UV-Vis spectrophotometric (UV-1800, Shimadzu, Kyoto, Japan) method. A calibration curve was prepared by dissolving PraNa powder in water to create standard solutions in the range of 5–25 μg/mL, with absorbance measured at 238 nm (R^2^ = 0.999). Preliminary trials were conducted to assess whether specific tablet excipients, such as iron oxide, sodium stearyl fumarate, or PVP, interfered with PraNa quantification at the selected wavelength.

The quantification of PraNa in the tablets was performed by dispersing each tablet in 900 mL of water. After two hours of mechanical stirring, the absorbance was measured, and the PraNa content was determined using the calibration curve. The test was conducted on five tablets for each type of PraNa tablet.

### 2.4. Preparation of Thickened Water Containing PraNa Tablets

Pensa PraNa tablet (weighing 0.149 ± 0.003 g) was selected to be dispersed in 5 g of thickened water, resulting in a total quantity of 5.15 g. This amount is suitable for administration using a teaspoon and represents the single dose to be administered (S_D).

All batches were prepared by pulverizing the tablets in a mortar with a pestle and then dispersing the powder into RU_TL and EP_TL under manual agitation for 5 min. Each batch consisted of 5 tablets and 25 g of thickened water.

### 2.5. Content Uniformity for Thickened Water Containing PraNa Tablets

The content uniformity of the gelled samples prepared with Pensa tablets was evaluated similarly to that of the tablets (Section 2.3) by dispersing an S_D in 900 mL of water and analyzing the PraNa content after two hours of mechanical stirring. The quantification of PraNa was performed as described for the tablets, except for samples containing the preservative potassium sorbate, which interferes with UV-based determination. In such cases, a dual-wavelength spectrophotometric method was used, measuring absorbance at 238 nm and 259 nm. This method was previously applied for PraNa quantification in the presence of parabens [19].

### 2.6. Rheological Analysis

The rheological analyses were performed using a stress-controlled rotational rheometer (Kinexus lab+, Malvern, UK) using a C40/4 cone-plate geometry on RU_TL and EP_TL, both with and without the presence of dispersed Pensa PraNa tablets at 25 and 37 °C. All samples were subjected to small amplitude oscillation tests.

Stress sweep analysis was conducted by applying a stress from 0.05 to 100 Pa at a frequency of 1 Hz. The linear viscoelastic region (LVR) limit was determined as the stress value at which the complex modulus (G*) deviated by 10% from the plateau value [20].

A frequency sweep test was then performed by increasing the oscillation frequency from 0.01 to 1 Hz while maintaining a constant stress within the LVR (1 Pa).

All the samples were analyzed in triplicate.

### 2.7. Dissolution Studies of Thickened Water Containing PraNa Tablets

The dissolution studies were carried out in thickened waters prepared with Pensa PraNa tablets using a dissolution apparatus II (AT7 Smart, Sotax, Aesch, Switzerland) with 900 mL of deionized water as the dissolution medium, maintained at 37 °C. The paddle rotation speed was set to 50 rpm. A specific amount (the S-D) of thickened water containing the dissolved PraNa tablets was introduced into the bottom of the dissolution vessel using a modified syringe with a cut tip. Samples were withdrawn at 0, 5, 15, 30, 60, 90, and 120 min. If the PraNa dissolution was incomplete at 120 min, the test was extended up to 480 min. Considering that the solubility of PraNa is higher than 10 mg/mL [21], all dissolution tests are conducted under sink conditions.

The PraNa quantification was performed as described in Section 2.3, or in Section 2.5 for samples containing potassium sorbate [19].

Additional studies were conducted only on selected RU_TL and EP_TL samples prepared using double or half the amount of Pensa PraNa tablets or prepared with PraNa tablets from other brands (PraNa Ratiopharm and EG tablets). In these additional studies, the weight of a single dose was adjusted based on the weight of the tablets used to prepare the loaded gel, ensuring it contained a PraNa dosage equivalent to 40 mg (2 Pensa tablets), 10 mg (half a Pensa tablet), or 20 mg (Ratiopharm and EG tablets).

## 3. Results and Discussion

### 3.1. Content Uniformity of Tablets and Thickened Water

The tablets used in this study had a weight of approximately 150 mg (Pravastatina Pensa). Across all tablet batches, the maximum percentage deviation of a single unit from the average weight was 2.5%, well below the European Pharmacopoeia (Ph. Eur.) limit of 7.5%, confirming that all these commercial products comply with Ph. Eur. standards [22]. The average amount of PraNa in the tablets ranged from 20.1 to 21.4 mg, compared to the nominal dose of 20 mg. In all cases, the PraNa content was compliant with Ph. Eur. requirements [23].

The thickened waters loaded with Pensa PraNa tablets were analyzed in terms of PraNa concentration and of PraNa amount administered using a single dose (S-D) (Table 3). Across the different preparations, the PraNa concentration ranged from 3.51 to 4.34 mg/g, while the amount of PraNa administered with an S-D (5.15 g) ranged from 18.5 to 22.3 mg. In all cases, variability within each prepared thickened water was low, with the highest coefficient of variation being approximately 6% with an average value of 2.7% (for the PraNa tablets it was equal to 2.6%). The standard deviation in the PraNa amount between tablets and gelled beverages was comparable for the majority of the samples. Additionally, the maximum percentage deviation in PraNa administered between tablets and all gelled products was 13%, while the average deviation, calculated as absolute values, was 6.3%. These results suggest that thickened water loaded with tablets is a viable alternative method for tablet administration, ensuring consistent quality independent by the jellified system used.

### 3.2. Rheological Behaviour

All the jellified vehicles, both with and without dispersed PraNa tablets, were compared in terms of rheological behaviour at 25 °C and 37 °C.

Initially, the samples were subjected to oscillation stress sweep analysis to identify the linear viscoelastic region (LVR), defined as the range of stress values within which the measured moduli remain independent of the applied stress. The samples exhibited typical stress sweep test profiles, with a constant complex modulus up to the LVR limit. The LVR limit values ranged from approximately 1.2–1.5 Pa for HyF grade 1 at both temperatures, to around 20–30 Pa for ThUp grade 3. Following this analysis, a stress value of 1 Pa was selected to perform the frequency sweep tests of all the samples.

**Figure 1:**
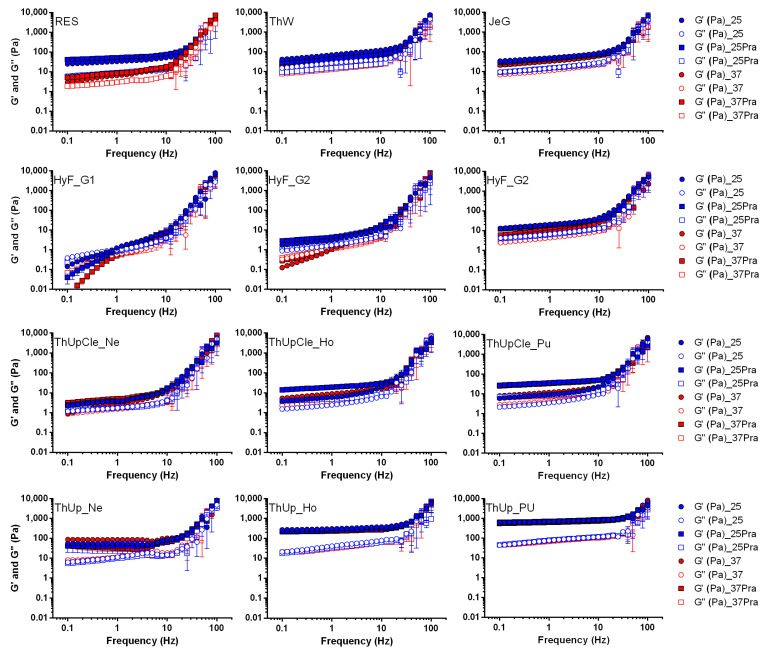
Frequency sweep tests for all jellified vehicles with and without pravastatin sodium at temperature of 25 and 37 °C. Thickened waters containing pravastatin were prepared with Pensa PraNa tablets.

The results of the frequency analysis (moduli vs. frequency) are reported in Figure 1 (data of viscosity are reported in Figure S1 of the Supplementary Materials). Comparing the traces with the typical viscoelastic spectrum [24] reveals that most samples align with the rubbery plateau at lower frequencies and the transition region at higher frequencies, reflecting the expected behaviour within these zones. At lower frequencies, the samples exhibit a behaviour characteristic of a strong gel, with G′ exceeding G′′ and both moduli remaining nearly independent of frequency. As the frequency increases beyond certain thresholds (ranging from 10 to 30 Hz, depending on the sample), both moduli increase, and G′′ surpasses G′, indicating a transition toward rubbery, solid-like behaviour. The only exception to this behaviour is observed in grade 1 of HyF. In this case, at frequencies below 1 Hz, the material exhibits a behaviour typical of the terminal region of the viscoelastic spectrum where the moduli are low, with G′′ exceeding G′, and both are highly frequency-dependent. This behaviour is characteristic of weak gels, with a crossover point, where the moduli invert, occurring around 1 Hz. A similar behaviour is partially observed for grade 2 of the same product, but only in tests conducted at 37 °C.

Considering the absolute values of the moduli in the lower frequency region (characteristic of a gel under normal conditions of use), the samples can be divided into three distinct groups. Res, ThW, JeG, grades Ho and Pu of ThUpCle, ThUp_Ne, and grade 3 of HyF are characterized by moduli in the range of 3–40 Pa and complex viscosity between 3 and 10 Pa·sec. In contrast, HyF grades 1 and 2, as well as ThickenUp_Ne, have moduli lower than 3 Pa and complex viscosity below 1 Pa·sec. On the other hand, the higher grades of ThUp (Ho and Pu) show moduli higher than 20–30 Pa (with G′ reaching values well above 100 Pa) and complex viscosity greater than 40 Pa·sec. Among all the samples, only the Resource sample showed a certain temperature dependency, while the presence of PraNa tablets did not significantly affect the rheology results. Clearly, the classification based on the frequency results is not absolute, and most samples exhibit their own specific behaviour. However, it is evident that HyF at the lowest grades stands out markedly from all other products due to its lower consistency, while the highest grades of ThickenUp are distinguished by their highest consistency.

### 3.3. Dissolution Studies

#### 3.3.1. Effect of Gelled Vehicle

The Pensa tablets, as well as the gelled vehicles containing the Pensa tablets, were analyzed in terms of dissolution performance to evaluate potential effects related to the composition or consistency of the gels.

Comparing the dissolution rates of tablets and gels is not straightforward, and no official guidelines exist for this specific comparison. However, in this case, since the tablets are designed for immediate-release, the gels could be considered almost equivalent if they achieve a comparable immediate-release. For this reason, it was decided to classify the release from a gelled vehicle as immediate if at least 75% of PraNa is released within the first 15 min, based on one of the acceptance criteria suggested by EMA in the Reflection paper on the dissolution specification for generic solid oral immediate-release products with systemic action [25]. This criterion is not intended as a measure of bioequivalence but rather as a tool to identify gelled vehicles that significantly alter the drug’s release kinetics.

**Figure 2:**
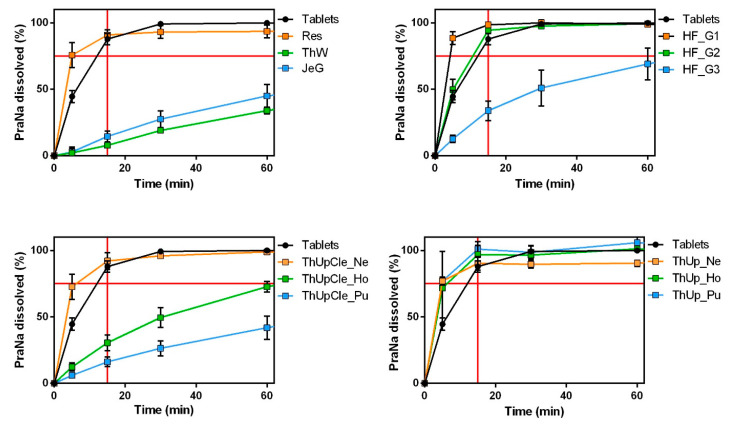
The PraNa release profiles for the tablets (Pensa) and all the jellified vehicles. The red lines on the x and y axes indicate a time of 15 min and a release of 75%, respectively.

The dissolution profiles of all gelled products are shown in Figure 2. Significant variability among the gelled vehicles is immediately apparent. The vehicles thickened with ThUp exhibited almost uniform results in terms of PraNa release, delivering the drug with immediate-release kinetics (lower right panel of Figure 2) despite having very different consistencies. In contrast, the other vehicles available in different grades (ThUpCle and HF) displayed markedly different PraNa release patterns depending on their consistency levels. Only those with lower consistencies achieved immediate-release profiles comparable to the original tablets. Finally, the three vehicles RU_TL Res, ThW, and JeG demonstrated distinctly different release behaviours, with only Res ensuring immediate-release. This highlights a contrasting situation: for ThUp-based products, consistency does not play a role (even though ThUp Pu has the highest consistency among all the gels tested), whereas for the other vehicles, the release profiles appear to depend on the gel’s composition (thickening agents) and/or its consistency.

These results can be explained by considering the thickening agents used in the different products. ThUp is the only product containing starch as a gelling agent and is specifically based on corn starch (although the label indicates corn starch, according to Methacanon et al. [9], it is a modified corn starch). In contrast, all the other products consist of blends of gums (guar gum and xanthan gum) or gum-like thickeners (pectin and carrageenan). Starch-based gels are reported to have low thickening power and a low gelling tendency. Moreover, their products often exhibit a grainy or pulpy texture and an opaque appearance. On the other hand, gum- and gum-like-thickened products generally possess higher viscosity (depending on the type and grade of the gum), with a smoother, slippery, or slimy mouthfeel and a transparent appearance [9].

A slowing drug-release effect for gum-based vehicles has been reported since the 1980s by Sarisuta and Parrott [26], although their studies did not aim to evaluate the potential for administering tablets using a gelled vehicle. Manrique et al. reported a reduction in the release rate of several drugs when crushed tablets were dispersed in gum-based vehicles [16,17]. The only study comparing starch- and gum-based formulations is that of Ilgaz et al., which demonstrated that the gelling vehicle significantly influences the drug’s dissolution rate, especially in thickened samples prepared with xanthan gum [11]. The findings of Ilgaz et al. partially align with those of this study. Specifically, the authors reported that the starch-based thickener exhibited a lower reduction in drug release compared to gum, although the effect depended on the drug’s solubility. Notably, starch did not influence drug release kinetics when the drug had low solubility. It is important to highlight that Ilgaz et al. used crushed tablets as a control instead of whole tablets, which could strongly affect the comparison (the dissolution rate of a powder is undoubtedly higher than that of whole tablets). However, to clarify whether the specific release profiles observed for ThUp are exclusively due to the starch-based thickener, further experiments were conducted using Pensa PraNa tablets and another commercially available starch-based thickener, Gel M (in this case, the thickener is a modified potato starch). The results of the dissolution study, reported in Figure S2 in the Supplementary Materials, confirm that the use of starch-based thickeners does not remarkably alter the dissolution profiles of dispersed tablets.

**Figure 3:**
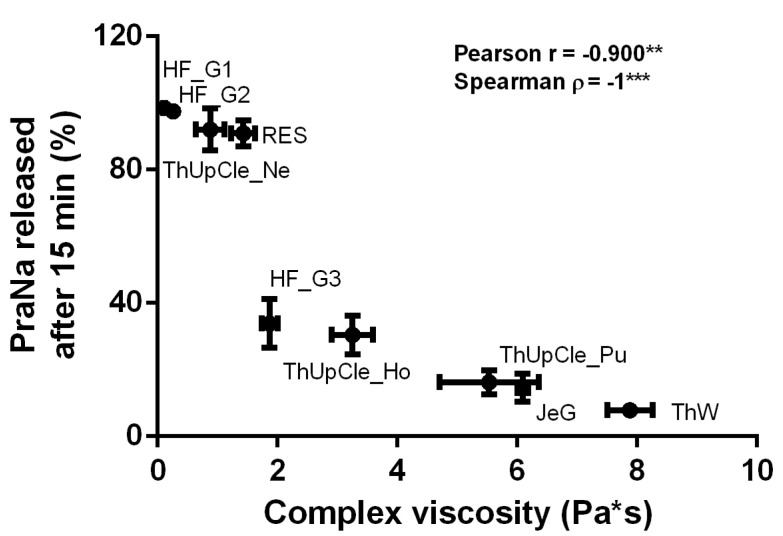
The correlation between the complex viscosity (measured at 1 Hz) of each gum-based vehicle and the amount of PraNa released at 15 min. The asterisks on the Pearson and Spearman coefficients indicate *p*-values between 0.01 and 0.001 (**) or lower than 0.001 (***).

Concerning the gum-based vehicles, it is difficult to determine from the literature whether the differing release profiles observed in the present study are attributable to the specific thickening agents used or the viscosity of the samples. For this reason, a correlation analysis between the complex viscosity (measured at 1 Hz) of each gum-based vehicle and the amount of drug released at 15 min was performed. The results, reported in Figure 3, show a strong and statistically significant correlation based on Pearson correlation analysis, indicating a linear trend in the data. However, when the correlation was assessed using Spearman’s correlation (based on the monotonic relationship between the two variables), the correlation coefficient was 1, indicating a perfect correlation. These results clearly indicate that, for the gum-based vehicles, the release profiles depend on the gel consistency, almost independently of the specific thickeners used.

#### 3.3.2. Effect of PraNa Dose

**Figure 4:**
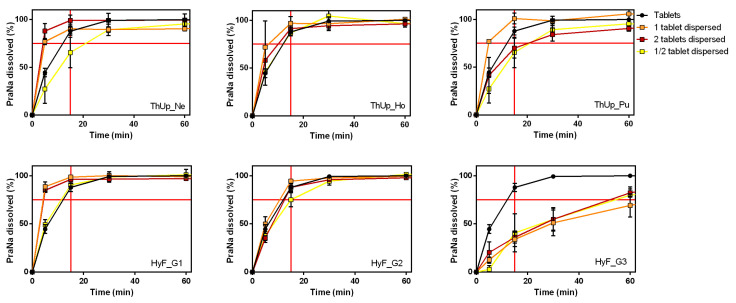
Effect of addition of 2 two tablets or half tablets on PraNa release from three different grades of ThUp (**upper panel graphs**) and HyF (**lower panel graphs**).

The use of the gelled vehicles for PraNa administration could be applied not only for the standard dose of 20 mg (a single tablet) but also for personalized dosing. In the case of tablets, a personalized dose can be achieved by administering multiple tablets or using a portion of a single tablet. To verify any effects caused by increasing the amount of powder dispersed or dissolved in the vehicles, PraNa-loaded thickened water was prepared using either two Pensa PraNa tablets or half a tablet, simulating doses of 40 mg and 10 mg, respectively (10, 20, and 40 mg are all doses of PraNa usually administered [27]). Three different grades of a starch-based product (ThUp) and a gum-based product (HyF) were selected as vehicles The homogeneity data for PraNa in these gelled vehicles are reported in Table S1 in the Supplementary Materials. The dissolution results are reported in Figure 4. As evident, doubling or halving the dose yields almost identical results, indicating that dose personalization does not significantly affect the release kinetics.

#### 3.3.3. Effect of PraNa Formulation

PraNa is a drug with an expired patent, and several equivalent alternatives (generic medicinal products) are available on the market. All these generic products differ exclusively in terms of excipients. ThUp and HyF gelled waters at three different grades were used to test the effect of different formulations dispersed in the vehicle, comparing two medicinal product equivalents to Pensa PraNa tablets: PraNa Ratiopharm and PraNa EG. PraNa Ratiopharm consists of uncoated tablets, like the Pensa version, although its formulation is more complex due to the presence of additional diluents/binders and two superdisintegrants (crosspovidone and croscarmellose sodium). On the other hand, PraNa EG has a composition similar to PraNa ratiopharm but is formulated as coated tablets (water-soluble coating with hypromellose). The homogeneity data for PraNa in these gelled vehicles are reported in Table S1 in the Supplementary Materials.

**Figure 5:**
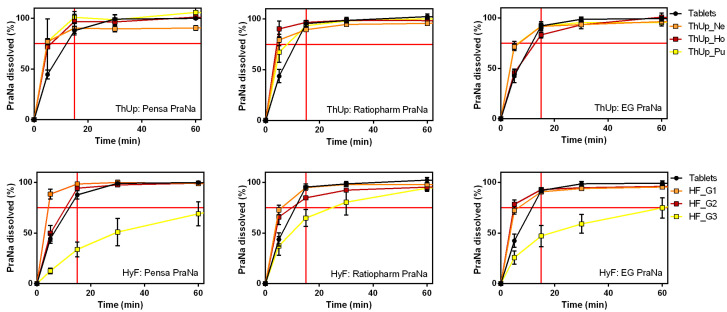
The effect of different tablet formulations on PraNa release from three grades of ThUp (**upper panel**) and HyF (**lower panel**). The graphs on the right show PraNa release from gelled water prepared with Pensa tablets, the middle graphs show release from gelled water prepared with Ratiopharm tablets, and the graphs on the left show release from gelled water prepared with EG tablets.

The release profiles of Pensa PraNa, Ratiopharm PraNa, and EG PraNa tablets, as reported in Figure S3 of the Supplementary Materials, reveal that all three medicinal products exhibit nearly identical dissolution behaviour. When the Ratiopharm and EG PraNa tablets were incorporated into the three grades of ThUp and HyF gels, the dissolution profiles (Figure 5) were almost identical to those obtained with the PraNa tablets. This clearly indicates that the release kinetics are primarily determined by the composition and viscosity of the vehicle, while the effect of the formulation itself is practically negligible.

## 4. Conclusions

Thickened water products that are commercially available exhibit a wide range of consistencies, and in many cases, there is no perfect match between different brands or even between different products from the same brand. Among the tested products, those thickened with starch-based additives demonstrated higher consistency compared to those prepared with gum-based gelling agents. The in vitro release profiles varied significantly among the analyzed products. The main differences were attributed to the type of thickening agent used. Specifically, in the presence of starch-based thickeners, all gelled water samples exhibited nearly identical pravastatin release kinetics, closely resembling the immediate-release profiles of the original tablets. Conversely, the thickened waters prepared with gum-based gelling agents showed substantial variability in pravastatin release profiles, which were strongly correlated with their viscosity values. Changes in the amount of crushed tablets added or variations in tablet formulations did not significantly alter pravastatin release kinetics, indicating that the type and/or viscosity of the thickened water primarily governs drug release.

The results of this study demonstrated that starch-based gelled waters have the least impact on drug release. From this perspective, they represent the best option for administering solid oral dosage forms manufactured as immediate-release tablets. On the other hand, it is well-known that gum-based products are usually preferred by patients due to their better mouthfeel and transparent appearance. Therefore, developing products that combine both types of thickening agents in optimal proportions could offer acceptable drug release while maintaining good patient compliance.

It is important to emphasize that the results obtained in this study are specifically valid for drugs with high solubility, such as sodium pravastatin (a BCS Class III drug). The release behaviour observed in thickened water formulations may not be directly applicable to drugs with lower solubility, such as those in BCS Classes II and IV. These drugs often exhibit different solubility and release profiles due to their distinct physicochemical properties, which can significantly alter their interaction with the jelled vehicle. Consequently, caution should be exercised in generalizing these findings to drugs with limited solubility, as their behaviour in similar formulations could differ substantially. Future studies focusing on these drug classes are necessary to fully understand the effects of thickened water on their release dynamics.

## Figures and Tables

**Table 1:** General information and abbreviations of all thickened water products used in manuscript.

Product Type	Product Name	Manufacturer	Thickening Agents	Grades Available	Abbreviation
**Ready-to-use thickened liquids (RU_TL)**	**Thickened water sweet fruit taste**	Nutrisens	Guar gum, pectin xanthan gum and calcium lactate	1	ThW
	**Gelly’Gel sweetened**	Nutrisens	Carrageenan and xanthan gum	1	JeG
	Hydra’Fruit *	Nutrisens	Xanthan gum, guar gum and pectin	3 *	HyF_G1 HyF_G2 HyF_G3
	**Resource bevanda gelificata**	Nestlè	Carrageenan, xanthan gum and guar gum	1	Res
**Powders for the preparation of extemporaneous thickened liquids (EP_TL)**	**Resource ThickenUp**	Nestlè	Corn starch	All **	ThUp_Ne ThUp_Ho ThUp_Pu
	**Resource ThickenUp Clear**	Nestlè	Maltodextrin, xanthan gum	All **	ThUpCle_Ne ThUpCle_Ho ThUpCle_Pu

* The Hydra’Fruit products are labelled and described in the brochure as having consistencies classified as grade 1 (semiliquid), grade 2 (creamy), and grade 3 (semisolid). However, the meaning of these classifications is unclear, as they do not precisely align with the standard definitions proposed by the National Dysphagia Diet (NDD classification) or the International Dysphagia Diet Standardisation Initiative (IDDSI levels). ** Since the product is available in powder form, all consistencies can be achieved by adjusting the amount of powder used. The prepared consistencies recommended by the manufacturer align with the DDS classification, specifically nectar (Ne), honey (Ho), and pudding (Pu).

**Table 2:** The concentrations of ThUp and ThUpCle used for the preparation of the different grades according to the label information (following the NDD classification).

Consistency Grades	ThUpCle		ThUp	
	g/100 mL	% *w*/*w*	g/100 mL	% *w*/*w*
**Nectar**	1.2	1.2	4.5	4.3
**Honey**	2.4	2.3	6.8	6.3
**Pudding**	3.6	3.5	9.0	8.3

**Table 3:** Pravastatin sodium concentration and amount administered in single dose (5.15 g) of thickened water prepared with Pensa PraNa tablets.

Product Type	Product Name	PraNa Concentration (mg/g)	PraNa Amount in the Single Dose (mg)
**Ready-to-use thickened liquids (RU_TL)**	**ThW**	4.34 ± 0.06	22.3 ± 0.3
	**JeG**	4.25 ± 0.15	21.9 ± 0.8
	**HyF_G1**	3.93 ± 0.10	20.2 ± 0.8
	**HyF_G2**	3.71 ± 0.09	19.1 ± 0.5
	**HyF_G3**	3.75 ± 0.11	19.3 ± 0.6
	**Res**	4.05 ± 0.25	20.8 ± 1.3
**Powders for the preparation of extemporaneous thickened liquids (EP_TL)**	**ThUp_Ne**	3.59 ± 0.09	18.5 ± 0.5
	**ThUp_Ho**	3.77 ± 0.09	19.4 ± 0.5
	**ThUp_Pu**	3.51 ± 0.11	18.1 ± 0.6
	**ThUpCle_Ne**	3.96 ± 0.03	20.4 ± 0.2
	**ThUpCle_Ho**	3.79 ± 0.07	19.5 ± 0.4
	**ThUpCle_Pu**	3.99 ± 0.10	20.6 ± 0.5

## Data Availability

The original contributions presented in this study are included in the article/Supplementary Material. Further inquiries can be directed to the corresponding author.

## Supplementary Materials

The following supporting information can be downloaded at: https://www.mdpi.com/article/10.3390/pharmaceutics17020251/s1, Figure S1. Complex viscosity of jellified vehicles (Panel A: RU_TL; Panel B: EP_TL) with and without Pravastatin sodium at 25 °C and 37 °C. The complex viscosity is presented as power-law viscosity, determined by analyzing the complex viscosity vs. frequency curves using a standard power-law model. The thickened water containing Pravastatin was prepared using Pensa PraNa tablets; Figure S2. PraNa release profiles from the tablets (Pensa) and jellified vehicles prepared with the powders for the preparation of extemporaneous thickened liquids Gel M (Nutrisens Medical, France). The red lines on the x and y axes indicate a time of 15 min and a release of 75%, respectively; Figure S3. PraNa release profiles from the tablets of the three different generic medicinal products The red lines on the x and y axes indicate a time of 15 min and a release of 75%, respectively; Table S1. Pravastatin sodium concentration and amount administered in a single dose of thickened water (Hydra’Fruit and Resource ThickenUp at all grades), prepared with two tablets or half a tablet of Pensa PraNa, or with a single tablet of PraNa Ratiopharm or PraNa EG.
