# Supplementary material for: Toward the Optimal Choice of Gelled Vehicles for Oral Drug Administration in Dysphagic Patients
Source: Pharmaceutics. 2025 Feb 14;17(2):251. doi: 10.3390/pharmaceutics17020251 (PMC11860041; doi:10.3390/pharmaceutics17020251)
Supplement: Supplementary file 1 [file pharmaceutics-17-00251-s001.zip › pharmaceutics-3419134-supplementary.pdf]

Supplementary material of

Toward the Optimal Choice of Gelled Vehicles for Oral Drug Administration in Dysphagic Patients

Serena Logrippo [1,2], Roberta Ganzetti [3], Matteo Sestili [4], Diego Romano Perinelli [5] , Marco Cespi [5,*] and Giulia Bonacucina [5]

1 Hospital Pharmacy, Santa Maria della Stella Hospital, USL Umbria 2, 05018 Orvieto, Italy

2 Hospital Pharmacy, Engles Profili Hospital, AST Ancona, 60044 Fabriano, Italy

3 Hospital Pharmacy, Carlo Urbani Hospital, AST Ancona, 60035 Jesi, Italy

4 Territorial Pharmaceutical Service, AST Ancona, 60035 Jesi, Italy

5 School of Pharmacy, University of Camerino, CHIP Building via Madonna delle Carceri, 62032 Camerino, Italy; diego.perinelli@unicam.it (D.R.P.); giulia.bonacucina@unicam.it (G.B.)

* Correspondence: marco.cespi@unicam.it

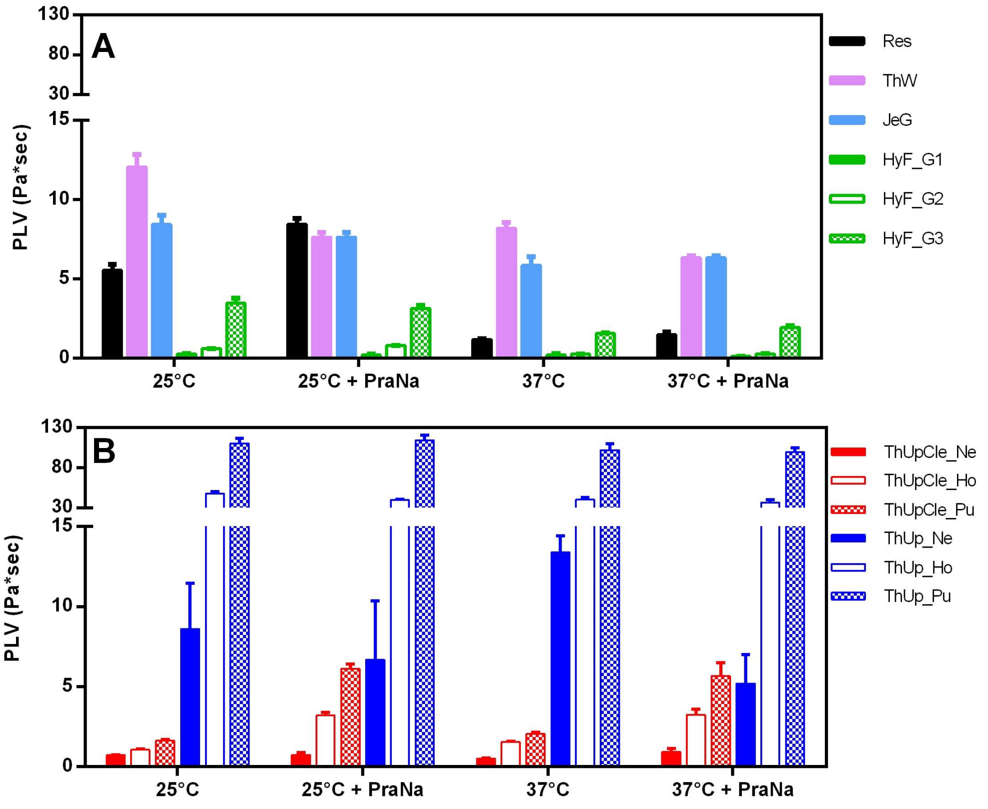

Figure S1. Complex viscosity of jellified vehicles (Panel A: RU_TL; Panel B: EP_TL) with and without Pravastatin sodium at 25°C and 37°C. The complex viscosity is presented as power-law viscosity, determined by analyzing the complex viscosity vs. frequency curves using a standard power-law model. The thickened water containing Pravastatin was prepared using Pensa PraNa tablets.

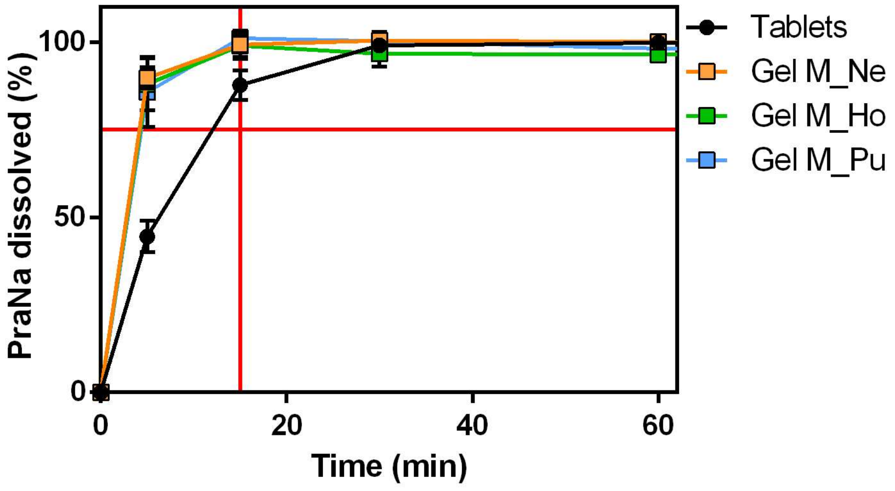

Figure S2. PraNa release profiles from the tablets (Pensa) and jellified vehicles prepared with the powders for the preparation of extemporaneous thickened liquids Gel M (Nutrisens Medical, France). The red lines on the x and y axes indicate a time of 15 minutes and a release of 75%, respectively.

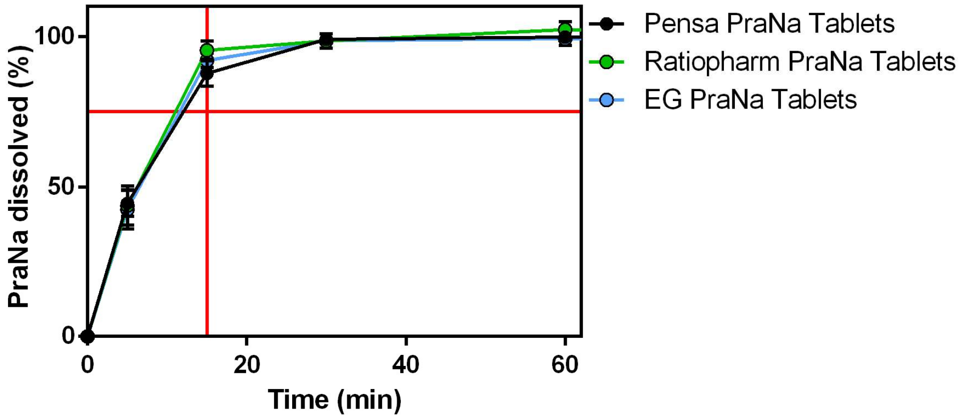

Figure S3. PraNa release profiles from the tablets of the three different generic medicinal products The red lines on the x and y axes indicate a time of 15 minutes and a release of 75%, respectively.

Table S1. Pravastatin sodium concentration and amount administered in a single dose of thickened water (Hydra'Fruit and Resource ThickenUp at all grades), prepared with two tablets or half a tablet of Pensa PraNa, or with a single tablet of PraNa Ratiopharm or PraNa EG.

Tickened water	Tablet used	N° of tablets in the single dose	PraNa concentration (mg/g)	PraNa amount in the single dose (mg)
HyF_G1	Pensa	2	7.51±0.07	39.8±0.35
HyF_G2	Pensa	2	7.36±0.18	39.0±0.95
HyF_G3	Pensa	2	7.57±0.12	40.1±0.61
ThUp_Ne	Pensa	2	6.89±0.03	36.5±0.16
ThUp_Ho	Pensa	2	7.40±0.14	39.2±0.76
ThUp_Pu	Pensa	2	6.56±0.18	34.8±0.97
HyF_G1	Pensa	1/2	2.15±0.14	10.9±0.72
HyF_G2	Pensa	1/2	1.92±0.03	9.7±0.17
HyF_G3	Pensa	1/2	2.01±0.07	10.2±0.34
ThUp_Ne	Pensa	1/2	1.82±0.07	9.3±0.33
ThUp_Ho	Pensa	1/2	2.00±0.07	10.1±0.37
ThUp_Pu	Pensa	1/2	2.15±0.08	10.9±0.39
HyF_G1	EG	1	4.09±0.06	21.3±0.33
HyF_G2	EG	1	4.19±0.07	21.8±0.38
HyF_G3	EG	1	4.11±0.11	21.4±0.56
ThUp_Ne	EG	1	3.68±0.08	19.1±0.42
ThUp_Ho	EG	1	3.69±0.15	19.2±0.80
ThUp_Pu	EG	1	3.65±0.12	19.1±0.65
HyF_G1	Ratiopharm	1	4.02±0.03	20.9±0.17
HyF_G2	Ratiopharm	1	3.83±0.08	19.9±0.42
HyF_G3	Ratiopharm	1	4.02±0.08	20.9±0.42
ThUp_Ne	Ratiopharm	1	3.84±0.03	20.0±0.16
ThUp_Ho	Ratiopharm	1	3.73±0.09	19.4±0.45
ThUp_Pu	Ratiopharm	1	3.62±0.17	18.8±0.90
